# Expression profile and down-regulation of argininosuccinate synthetase in hepatocellular carcinoma in a transgenic mouse model

**DOI:** 10.1186/s12929-015-0114-6

**Published:** 2015-01-23

**Authors:** Shih-Chang Shiue, Miao-Zeng Huang, Ting-Fen Tsai, Alice Chien Chang, Kong Bung Choo, Chiu-Jung Huang, Tsung-Sheng Su

**Affiliations:** Institute of Microbiology & Immunology, National Yang-Ming University, Taipei, Taiwan; Department of Medical Research, Taipei Veterans General Hospital, Taipei, 112 Taiwan; Department of Life Sciences and Institute of Genome Sciences, National Yang-Ming University, Taipei, Taiwan; Institute of Neuroscience, National Yang-Ming University, Taipei, Taiwan; Department of Preclinical Sciences, Faculty of Medicine and Health Sciences, Universiti Tunku Abdul Rahman, Selangor, Malaysia; Centre for Stem Cell Research, Universiti Tunku Abdul Rahman, Selangor, Malaysia; Department of Animal Science, Chinese Culture University, Taipei, Taiwan; Graduate Institute of Biotechnology, Chinese Culture University, Taipei, Taiwan

**Keywords:** Argininosuccinate synthetase, Transgenic mouse model, Hepatocellular carcinoma, Embryo expression map, Brain expression map, Ventricular zone, Subventricular zone, Post-transcriptional regulation, Bacterial artificial chromosome, GFP reporter gene

## Abstract

**Background:**

Argininosuccinate synthetase (ASS) participates in urea and nitric oxide production and is a rate-limiting enzyme in arginine biosynthesis. Regulation of *ASS* expression appears complex and dynamic. In addition to transcriptional regulation, a novel post-transcriptional regulation affecting nuclear precursor RNA stability has been reported. Moreover, many cancers, including hepatocellular carcinoma (HCC), have been found not to express *ASS* mRNA; therefore, they are auxotrophic for arginine. To study when and where *ASS* is expressed and whether post-transcriptional regulation is undermined in particular temporal and spatial expression and in pathological events such as HCC, we set up a transgenic mouse system with modified BAC (bacterial artificial chromosome) carrying the human *ASS* gene tagged with an *EGFP* reporter.

**Results:**

We established and characterized the transgenic mouse models based on the use of two BAC-based *EGFP* reporter cassettes: a transcription reporter and a transcription/post-transcription coupled reporter. Using such a transgenic mouse system, EGFP fluorescence pattern in E14.5 embryo was examined. Profiles of fluorescence and that of *Ass* RNA in *in situ* hybridization were found to be in good agreement in general, yet our system has the advantages of sensitivity and direct fluorescence visualization. By comparing expression patterns between mice carrying the transcription reporter and those carrying the transcription/post-transcription couple reporter, a post-transcriptional up-regulation of *ASS* was found around the ventricular zone/subventricular zone of E14.5 embryonic brain. In the EGFP fluorescence pattern and mRNA level in adult tissues, tissue-specific regulation was found to be mainly controlled at transcriptional initiation. Furthermore, strong EGFP expression was found in brain regions of olfactory bulb, septum, habenular nucleus and choroid plexus of the young transgenic mice. On the other hand, in crossing to hepatitis B virus X protein (*HBx*)-transgenic mice, the *Tg (ASS-EGFP, HBx)* double transgenic mice developed HCC in which *ASS* expression was down-regulated, as in clinical samples.

**Conclusions:**

The BAC transgenic mouse model described is a valuable tool for studying *ASS* gene expression. Moreover, this mouse model is a close reproduction of clinical behavior of *ASS* in HCC and is useful in testing arginine-depleting agents and for studies of the role of ASS in tumorigenesis.

## Background

Argininosuccinate synthetase (ASS; EC 6.3.4.5) catalyzes the conversion of citrulline and aspartate to argininosuccinate, which is subsequently converted to arginine by argininosuccinate lyase. Arginine plays an important role in the synthesis of urea, nitric oxide (NO) and polyamines, among other metabolites [1]. In the process, ASS fine-tunes NO production to maintain cellular homeostasis in response to cellular and environmental stimuli. ASS is ubiquitously expressed but the highest enzyme activities are found in the urea cycle in the liver to eliminate ammonia [2,3].

Regulation of *ASS* expression is complex and dynamic. Hormones, including glucocorticoid, glucagon and insulin, are major regulators of the expression of urea cycle enzymes, including ASS, in the liver [2]. We have reported an upstream cAMP response element (CRE) targeted by the CRE-binding protein (CREB) to mediate glucagon action [4]. On the other hand, *ASS* expression in non-hepatic cells has been shown to be induced by interleukin-1β through NF-κB activation *via* a putative NF-κB binding site in the *ASS* promoter [5]. *ASS* gene expression also involves interactions between positive transcriptional factors c-Myc and Sp4 and negative factor HIF-1α in the proximal promoter [6]. Furthermore, we have described a novel post-transcriptional event in a canavanine-resistant variant of a human epithelial cell line that regulates the stability of *ASS* nuclear precursor RNA, resulting in elevated ASS activities [7,8]. In addition, our recent study showed that the formation of the 3′-end of the human *ASS* mRNA is modulated by a highly polymorphic GT microsatellite located downstream of the poly (A) signal [9]. The identified post-transcriptional regulation events may have physiological relevance and is an added mechanism for tight regulation of *ASS* expression. Of clinical significance is the finding that *ASS* transcription is stimulated by glutamine and repressed by arginine in mammalian tissue cultures [10–12].

A number of tumor types, including hepatocellular carcinoma (HCC), melanoma, prostate, pancreatic and renal cancers, clearly show down-regulated *ASS* expression and are auxotrophic to arginine [13–15]. In HCC, down-regulated *ASS* expression has been linked to clinicopathological features and post-resectional patient survival [16,17]. Loss of ASS expression has been exploited as a predictive biomarker for cancers including malignant pleural mesothelioma and epithelial ovarian tumor, to name a few [18,19]. Clinical applications of an arginine-depleting enzyme, such as arginine deaminase (ADI), to treat ASS-deficient tumors have been tested, opening up new venues for further development of auxotrophic cancer therapy [15].

**Figure 1:**
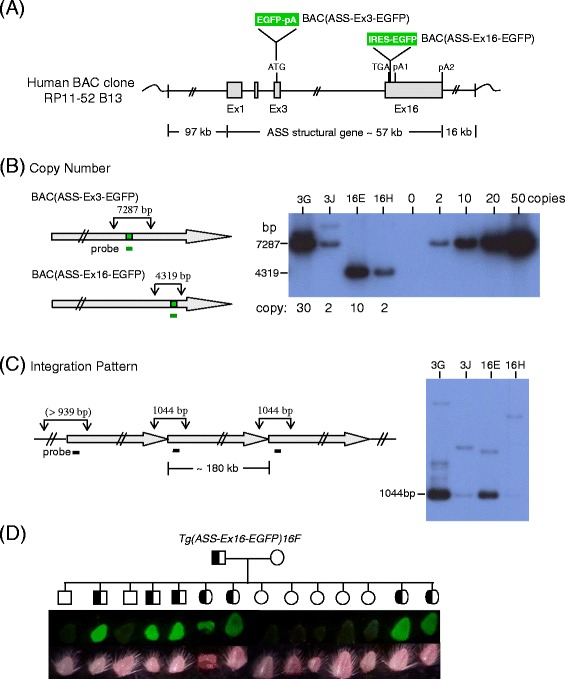
**Analysis of the transgene structure in the BAC (ASS-EGFP) transgenic mice. (A)** Overall view of BAC (ASS-EGFP) constructs showing relative positions of the *EGFP* transgene and the human *ASS* exons. Insertions of the *EGFP* gene with a polyadenylation signal into exon 3 (EGFP-pA) or an IRES-EGFP sequence into exon 16 of the *ASS* gene of BAC clone RP11-52B13 created the BAC (ASS-Ex3-EGFP) and BAC (ASS-Ex16-EGFP) constructs, respectively. The lengths of the 5′ and 3′ human genomic sequences included in the BAC construct and the *ASS* structural gene are shown. Wavy line at the end represents the vector sequence. **(B)** Determination of transgene copy number. Genomic DNAs from offspring of the second generation transgenic mice (F2) was digested with *Eco*RI and Southern blot analysis was performed using an *EGFP* probe. The horizontal grey arrows are schematic representation of the transgene; the green bars represent the *EGFP* sequence; downward arrows indicate the *Eco*RI sites shown with expected sizes of the hybridized *Eco*RI fragments. The signal of a BAC (ASS-Ex3-EGFP) DNA preparation with predetermined copy number was used to estimate the transgene copy number in the mouse lines. The abbreviated transgenic line designations 3G, 3 J and 16E, 16H were two different lines each from the BAC (ASS-Ex3-EGFP) and BAC (ASS-Ex16-EGFP) transgenes, respectively; the abbreviated designations are also used in other figures. **(C)** Confirmation of head-to-tail transgene integration. The *Pst*I sites (downward arrows) and the expected 1,044-bp head-to-tail hybridized fragments are shown. The size of junction fragment may be greater than 939 bp (>939 bp) which is determined by position of the next *Pst*I site in the mouse genome. **(D)** Monitoring of *ASS-EGFP* transgene transmission by fluorescence signals of tail samples. Pedigree analysis of the transgenic line *Tg (ASS-Ex16-EGFP) 16 F* is shown. Upper and lower rows were observed by dissecting microscopy under fluorescent light or white light, respectively.

To investigate transcriptional and post-transcriptional regulation of the *ASS* gene, we established a transgenic mouse system using a modified bacterial artificial chromosome (BAC) carrying the human *ASS* gene tagged with the enhanced green fluorescent protein (*EGFP*) reporter gene [20]. Two transgenic mouse lines were generated. One line was *Tg (ASS-Ex3-EGFP)* which carries the transcription reporter BAC (ASS-Ex3-EGFP) (Figure 1A), where *EGFP* was knocked-in at the initiation codon of the human *ASS* gene and *EGFP* transcription is terminated by a SV40 poly (A) signal. EGFP activities of *Tg (ASS-Ex3-EGFP)* mainly reflect promoter activities of the *ASS* gene. Another line *Tg (ASS-Ex16-EGFP)* carries the transcription/post-transcription couple reporter BAC (ASS-Ex16-EGFP) (Figure 1A), where *EGFP* with an internal ribosome entry site (IRES) is inserted into the terminal exon at site between the stop codon and the polyA signal of the *ASS* gene; in such a configuration, translation of *EGFP* in the bicistronic transcript is regulated by the IRES mechanism. EGFP activities thus expressed are subjected to both transcriptional and post-transcriptional regulation as that of the endogenous *ASS* mRNA. Using these transgenic mouse lines, we have taken liver, the organ for urea production, intestine and kidney that are responsible for arginine biosynthesis as a model for temporal and spatial expression analyses [20]. We found the expression of the *EGFP* reporter gene in the transgenic mice faithfully reproduced that of the endogenous gene, suggesting that sufficient *ASS* regulatory elements are included in the transgene. Moreover, comparison between the *EGFP* expression profiles of the two transgenic lines indicated the developmental and tissue-specific regulation is mainly controlled at the transcriptional level [20].

To demonstrate the merit of this transgenic mouse system in studying *ASS* gene expression, we further characterized the copy numbers and integration patterns of transgenes. We then examined the EGFP expression pattern in the embryo and in adult tissues, including the brain. Furthermore, to mimic clinical features of affected *ASS* gene expression in tumor tissues, the BAC (ASS-EGFP) transgenic mice were crossed to transgenic mice carrying the *HBx* gene of hepatitis B virus to induce HCC.

## Methods

### Animals

The mice were maintained in specific pathogen-free area of the animal holding facilities and were treated according to protocols approved by the Animal Care and Use Committee of the Taipei Veterans General Hospital. The transgenic mouse lines *Tg (ASS-Ex3-EGFP) Tsu* and *Tg (ASS-Ex16-EGFP) Tsu* have been deposited in the Rodent Model Resource Center, National Laboratory Animal Center, Taiwan, and are available for researchers upon requests.

### Construction of BAC (ASS-EGFP) reporters and generation of *Tg* (*ASS-EGFP)* transgenic mice

The human BAC clone, RP11-52B13, carrying ~170 kb of the human genome including the entire human *ASS* structural gene of ~57 kb and its 5′ and 3′ flanking sequences of 97 kb and 16 kb, respectively, was obtained from Genome Center, National Yang-Ming University, Taiwan. To construct the BAC transgene reporters, an *in vivo* recombination technique developed by Gust *et al*. [21] was used.

Purified BAC DNA was linearized by PI-*SceI* and was used in pronucleus microinjection of FVB/N fertilized eggs (Level Biotechnology Inc., Taiwan). Transgenic mice were identified by PCR detection of the *EGFP* sequence in tail DNA. The PCR forward primer, 5′-CGACCACTACCAGCAGAACAC-3′, was used for both lines while the reverse primers, 5′-GAGCAGACAGGCTGACAACC-3′ and 5′-AGGATGCTGGCTAGGATCG-3′, were for *Tg (ASS-Ex3-EGFP)* and *Tg (ASS-Ex16-EGFP)*, respectively. All founders were bred independently with wild-type FVB/N mice to obtain progenies.

### Generation of *Tg (ASS-Ex3-EGFP, HBx)* and *Tg (ASS-Ex16-EGFP, HBx)* double transgenic mice

A male transgenic mouse of the C57BL/6 strain carrying the hepatitis B virus X protein (*HBx*) transgene, i.e., *Tg (HBxA0106)* [22], was mated with female transgenic mice of the FVB/N strain of *Tg (ASS-Ex3-EGFP)* or *Tg (ASS-Ex16-EGFP)* to obtain double transgenic lines of *Tg (ASS-Ex3-EGFP, HBx)* and *Tg (ASS-Ex16-EGFP, HBx)*. Progenies carrying the *HBx* transgene were identified by PCR analysis of the presence of transgene sequence in tail DNA using primers 5′-CCTCCTTGGGCAACCTGTTCAG-3′ and 5′-ATGTGGCACTGAGGGACATGGC-3′. EGFP progenies were identified by PCR or by visualization of tail fluorescence of two week-old littermates under a fluorescence dissecting microscope.

### Southern blot analysis

DNA from mouse tails was purified using standard method. Tail DNA was digested with *Eco*RI or *Pst*I and subjected to Southern blot analysis using random primed-labeled *EGFP* probe or other probes as indicated in the text, and analyzed by autoradiography.

### Northern blot analysis

To isolate total RNA, tissues were grinded to powder in liquid nitrogen. The frozen powder was transferred to TRIzol reagent (Invitrogen, Carlsbad, CA) in a MagNA Lyser tube (Roche Applied Science, Indianapolis, IN) and was homogenized. Supernatant was obtained for RNA isolation following manufacturer’s instructions. For northern blot analysis, RNA was denatured in glyoxal before electrophoresis on a 1.2% agarose gel. RNA was transferred to Hybond N+ nylon membrane (GE Healthcare Life Sciences, UK), hybridized to an *EGFP* or an *ASS* cDNA probe and analyzed by a phosphorimager (Molecular Dynamics, Sunnyvale, CA).

### Gross anatomy and histochemical analysis

Mouse gross anatomy was performed following standard protocols. In brief, mouse was sacrificed by suffocation in a dry ice chamber. Images of internal organs after peritoneal wall incision or after gastrointestinal tract removal were acquired with a Canon PowerShot G11 digital camera built with blue filter (Sky-blue V/475 nm, Biotransman Ltd., Taiwan). For frozen sections, tissues collected were immersion in 4% buffered paraformaldehyde. After cryoprotected in graded sucrose solution, tissues were embedded in OCT compound (Tissue Tec, Sakara, Torrance, CA). Serial sections were collected from Leica cryostat (Leica Biosystems, Wetzlar, Germany) and mounted onto slides to examine EGFP expression by a fluorescence microscope (Olympus DP72, Japan), a confocal microscope (Nikon AIR Confocal System, Japan) or a digital slide scanner (Pannoramic Scan, 3DHISTECH Ltd., Hungary). Slides were counterstained with DAPI (4′,6-diamidino-2-phenylindole) (Roche Applied Science, Indianapolis, IN). For histochemical studies, tissues fixed in buffered paraformaldehyde and embedded in paraffin were deparaffinized, hydrated in graded ethanol and stained with hematoxylin and eosin (HE stain). For immunohistochemical studies, the tissue slides were first deparaffinized and antigen retrieved. After blocking, the slides were incubated overnight at 4°C with an anti-GFP rabbit polyclonal antibody (Chemicon) or with a mouse anti-ASS monoclonal antibody (BD Biosciences) both at 1:100 dilution. Subsequently, the slides were incubated with a biotinylated secondary antibody and streptavidin conjugated-HRP (horseradish peroxidase). The HRP was visualized by the application of substrate chromogen DAB (diaminobenzidine) (Dako, Glostrup, Denmark) to generate brown coloration where the slides were counterstained with hematoxylin [20].

## Results and discussion

### Generation and characterization of BAC (ASS-EGFP) transgenic mice

BAC (ASS-EGFP) transgenic mice were generated by pronuclear microinjection of FVB/N fertilized eggs with linearized transgene DNA. Mice harboring the transgene sequences were initially screened by PCR detection of the *EGFP* sequence in the genomic DNA of mouse tails. Among the 41 litters produced from two injections of BAC (ASS-Ex3-EGFP) (Figure 1A), 14 were EGFP-positive, and the mice are designated as *Tg (ASS-Ex3-EGFP).* Among the 39 litters produced from a single injection of BAC (ASS-Ex16-EGFP) (Figure 1A), 10 were EGFP-positive, and the mice are designated as *Tg (ASS-Ex16-EGFP).* Fourteen *Tg (ASS-Ex3-EGFP)* founders and 10 *Tg (ASS-Ex16-EGFP)* founders were independently bred with wild-type FVB/N mice. Transgenic mosaic mice and mice carrying multiple integration loci were not further studied here. On the other hand, mice carrying multiple transgene copies at a single integration site may have the advantages in providing better signal-to-noise fluorescence signals in immunochemical analyses of tissue sections [23]. Nevertheless, high transgene copy may result in aberrant regulation by bound and sequestering cellular *trans*-factors required for endogenous gene expression. Accordingly, transgenic mice harboring both high and low copy numbers were identified for subsequent investigation.

The transgene copy number and integration pattern were characterized by Southern blot analysis (Figure 1). Results showed that the two *Tg (ASS-Ex3-EGFP)* lines designated as *Tg (ASS-Ex3-EGFP) 3G and Tg (ASS-Ex3-EGFP) 3 J,* abbreviated as 3G and 3 J in Figure 1 and in subsequent figures, harbored ~30 and 2 copies of the transgene, and the two *Tg (ASS-Ex16-EGFP)* lines, *Tg (ASS-Ex16-EGFP) 16E* and *Tg (ASS-Ex16-EGFP) 16H*, 16E and 16H in the figures, harbored ~10 and 2 transgene copies, respectively (Figure 1B). In addition, *Tg (ASS-Ex16-EGFP) 16 F,* abbreviated as 16 F and carries 5 transgene copies (data not shown), was also used in subsequent studies. In the analysis of the transgene integration, transgenic lines with high or low copy number all showed the anticipated 1,044-bp junctional *Pst*I fragment of single head-to-tail integrations (Figure 1C). Minor spurious bands were noted in line 3G that could be due to minor rearrangements in the integrated transgenes, or occurrence of complex but minor transgene configurations. The transgene copy number and the configuration were stably transmitted in many generations of breeding (data not shown). Interestingly, *EGFP* transgene transmission was conveniently identified by direct visualization of fluorescence in the tails of littermates under a fluorescence dissecting microscope (Figure 1D), circumventing the tedious PCR-based identification of transgenic littermates.

### *ASS-EGFP* transgene expression in the embryo

**Figure 2:**
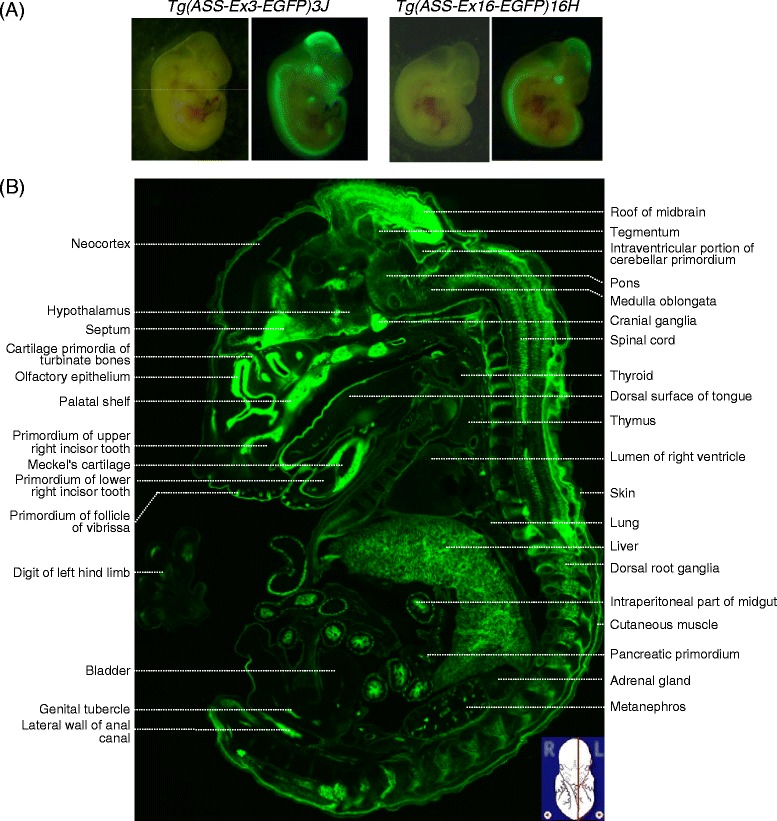
***ASS-EGFP*** **reporter gene expression in the embryo of the transgenic mice. (A)** Embryos of *Tg (ASS-Ex3-EGFP) 3 J* and *Tg (ASS-Ex16-EGFP) 16H* mice at E11.5 were examined under a fluorescence dissecting microscope. Images on the left and right were observed under white or fluorescent light, respectively. **(B)** Annotated expression pattern of E14.5 embryo of *Tg (ASS-Ex3-EGFP) 3G*. Serial frozen sagittal sections were collected and a representative section is shown. The sectional plane (red line) relative to the intact embryo is inserted in the bottom right corner where the embryo marking is adapted from GenePaint.org [26]. The structures are labelled based on Kaufman [27] and GenePaint.org [26].

When transgenic mouse embryos at embryonic day 11.5 (E11.5) was examined under a fluorescence dissecting microscope, particular structures that were presumably expressing ASS became visible (Figure 2A). For example, structures such as the brain and spinal cord show strong signals in both 3 J and 16H lines. In this respect, others have reported that mouse *Ass* mRNA is detectable as early as E1.0-E3.5 [24,25]. Conceivably, *ASS-EGFP* transgenic mouse is an ideal system to trace spatial and temporal *ASS* expression during embryonic development.

At E11.5, the EGFP expression pattern in the 3G line was similar to those of 3 J and 16H (data not shown). The 3G line, containing 30 transgene copies, is useful to reveal structures with weaker levels of expression. Moreover, there was no apparent sequestration of transcription factors to affect endogenous *Ass* gene expression using this line in our study of developmental regulation of liver, kidney and intestine [20]. Thus, 3G line was chosen to further analyze *ASS-EGFP* expression in the embryo. Serial frozen sagittal sections of E14.5 embryos of the 3G line were prepared and a representative section is shown in Figure 2B. Of note, autofluorescence of wild-type embryo was negligible when compared to EGFP signals in the 3G line (data not shown). Strong EGFP fluorescence was observed in structures including the olfactory epithelium, septum, midbrain, cranial ganglia, spinal cord, dorsal root ganglia, liver and the midgut. The pattern of ASS expression was similar to that of E14.5 embryos studied by RNA *in situ* hybridization [28]. However, the signal intensity of *Ass* RNA *in situ* is in general weak; tissues with low expression may become undetectable. Our transgenic mouse lines, thus, provide a sound model system to study *ASS-EGFP* expression *in vivo.*

**Figure 3:**
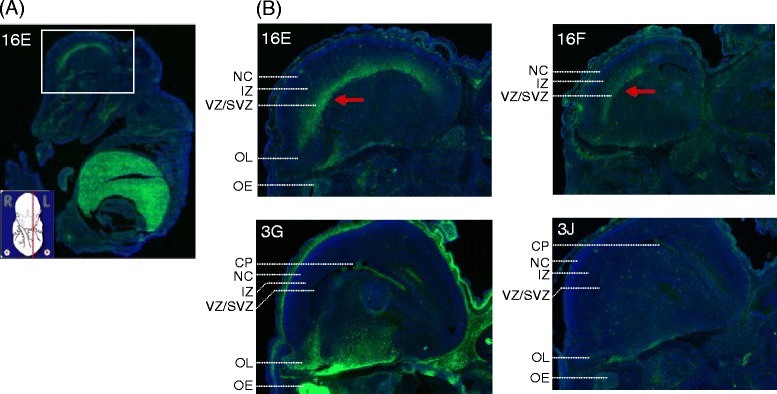
**Post-transcriptional up-regulation of** ***ASS*** **in cells around the ventricular zone/subventricular zone (VZ/SVZ) in E14.5 embryo of the** ***Tg (ASS-Ex16-EGFP)*** **line. (A)** A frozen sagittal image of E14.5 embryo from the 16E line is shown. The sectional plane (red line) relative to the intact embryo is inserted in the bottom left corner where the embryo marking is adapted from GenePaint.org. [26] **(B)** Comparable region (boxed field in A) from each mouse line was selected and magnified. Red arrow points to enhanced fluorescence signals. The abbreviated mouse designations are as described in the legend to Figure 1. The labels are as follows: CP, choroid plexus; IZ, intermediate zone; NC, neocortex; OE, olfactory epithelium; OL, olfactory lobe; VZ/SVZ, ventricular zone/subventricular zone.

To address the issue whether post-transcriptional regulation in *ASS* gene is in operation during embryonic development, *EGFP* expression patterns in E14.5 embryos of the *Tg (ASS-Ex16-EGFP)* line carrying the transcription/post-transcription couple reporter were studied. We found that the expression profiles were similar, yet significantly higher levels of EGFP expression were detected around the ventricular zone/subventricular zone (VZ/SVZ) of the brain in the *Tg (ASS-Ex16-EGFP)* line, i.e., 16E and 16 F, compared to that in the *Tg (ASS-Ex3-EGFP)* line*,* i.e., 3G and 3 J (Figure 3). The embryonic VZ/SVZ is the major site of neurogenesis of the mammalian telencephalon [29]. Our data suggest that there is post-transcriptional up-regulation of *ASS* in cells around VZ/SVZ. The mechanism and functional consequences of such a mode of regulation need to be further investigated.

### Expression of the *ASS-EGFP* transgene in adult organs

ASS protein is ubiquitously expressed, albeit with significant variations in the expression levels among tissues and organs [2]. We have previously studied EGFP expression patterns in the liver, kidney and intestine in both the *Tg (ASS-Ex3-EGFP)* and *Tg (ASS-Ex16-EGFP)* lines and found that EGFP signals faithfully reproduced the spatial and temporal expression patterns of the *ASS* gene [20].

**Figure 4:**
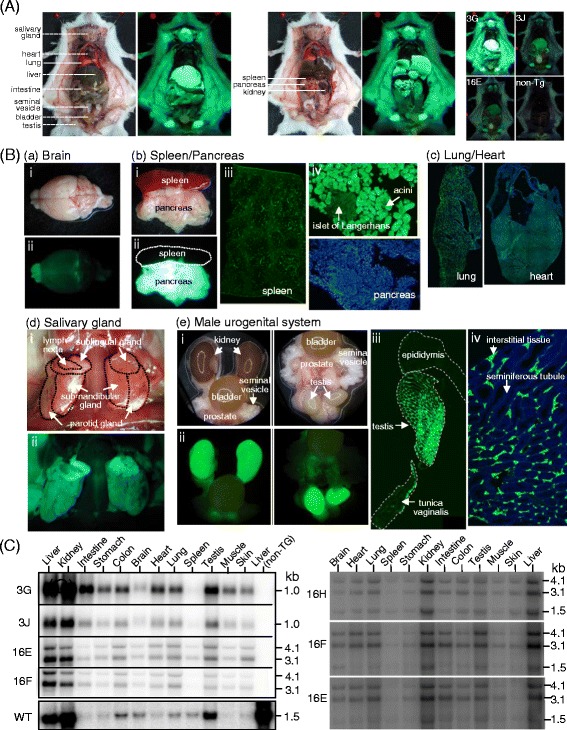
**Expression of the** ***ASS-EGFP*** **reporter gene in adult transgenic mice. (A)** Gross anatomy displaying EGFP fluorescence. Male mice of 3.5 month-old *Tg (ASS-Ex3-EGFP) 3G* line were sacrificed to display the internal organs. Images after peritoneal wall incision (left panel) and after gastrointestinal tract removal (middle panel) were acquired with a Canon PowerShot G11 digital camera built with blue filter. Fluorescence images were taken with an exposure time of 2 seconds. Right panel, comparison of fluorescence signals among the mouse lines. The abbreviated mouse designations are as described in the legend to Figure 1. non-Tg: a non-transgenic control. Images were taken with an exposure time of 4 seconds. **(B)** Study of EGFP fluorescence in (a) brain, (b) spleen and pancreas, (c) lung and heart, (d) salivary gland and (e) male urogenital system of the 3G line. Panels (i) and (ii) are whole-mount specimens examined under white and fluorescent light of a dissecting microscope, respectively. The rest of the panels were frozen sections examined by fluorescence microscopy. Top panel of (b) (iv) is a fluorescence image and the bottom one is that stained with DAPI. Panel (iv) of (e) is a higher magnification view of the testis counterstained with DAPI to show location of the interstitial tissue and seminiferous tubule. **(C)** Northern blot analysis. Left panel, detection of *EGFP* RNA expression in tissues of 3.5-month-old mice using an *EGFP* probe. The lane at the far right was RNA from a non-transgenic liver. The bottom lane was an analysis of *Ass* mRNA from a wild-type mouse using an *ASS* cDNA probe. Right panel, analysis of mouse *Ass* RNA and human *ASS* RNA in tissues of 27-day-old mice of the *Tg (ASS-Ex16-EGFP)* lines using an *ASS* cDNA probe. Lines 16H, 16 F and 16E carry 2, 5 and 10 transgene copies, respectively.

**Figure 5:**
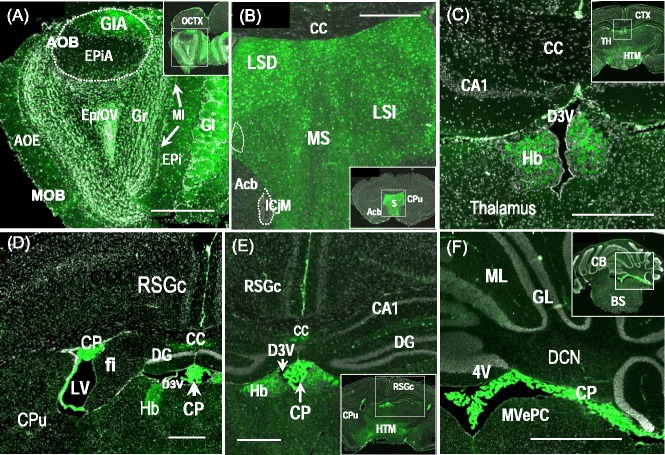
**Spatial expression of** ***ASS-EGFP*** **in the brain of a 4 week-old** ***Tg(ASS-Ex3-EGFP)3G*** **mouse.** Representative micrographs are shown. The insert of each micrograph indicates the location in brain where the image was taken. The scale represents 500 μm. **(A)** Granule neurons and cells in the glomerular layer in both major olfactory bulb (MOB) and accessory olfactory bulb (AOB) exhibiting strong EGFP expression. **(B)** In the septum region, significant EGFP expression is found in septal nuclei, i.e., LSD, LSI and MS. **(C)** In the thalamus, neurons of habenular nuclei are expressing EGFP whereas no significant EGFP expression was found in other nuclei of thalamus. Relatively weak EGFP expression was detected in the hypothalamus region. **(D)** Cells of choroid plexus in the lateral ventricle (LV) and the dorsal third ventricle (D3V) and **(E)** that in the dorsal third ventricle (D3V) exhibiting strong EGFP expression. **(F)** At the level of brain stem and cerebellum, cells of choroid plexus in the 4th ventricle (4V) exhibited strong EGFP expression. Abbreviations: 4V, 4th ventricle; Acb, nucleus accumbens; AOB, accessory olfactory bulb; AOE, anterior olfactory nucleus external; BS, brain stem; CA1, subregion of hippocampus; CB, cerebellum; cc, corpus callosum; CP, choroid plexus; CPu, caudate putamen; CTX, cerebral cortex; D3V, dorsal 3rd ventricle; DCN, deep cerebellar nuclei; DG, dentate gyrus; EPi, external plexiform layer; EPiA, external plexiform layer/AOB; Ep/OV, ependyma/olfactory ventricle; fi, fimbria; Gl, glomerular layer; GlA, glomerular layer/AOB; GL, granule layer; Gr, granule layer of olfactory bulb; Hb, habenular nucleus; HTM, hypothalamus; ICjM, islands of Calleja/major; LSD, lateral septal nucleus, dorsal part; LSI, lateral septal nucleus, intermediate part; LV, lateral ventricle; MI, mitral cell layer; MOB, major olfactory bulb; ML, molecular layer; MS, medial septal nucleus; MVePC, medial vestibular nucleus, parvicellular part; OCTX, orbital cortex; RSGc, retrosplenial granular cortex; S, septum; TH, thalamus.

To further investigate the global *ASS-EGFP* expression pattern in the transgenic mice, the gross anatomy of adult *Tg (ASS-Ex3-EGFP) 3G,* i.e. 3G, was studied where the salivary gland, liver, intestine, testis, pancreas and the kidney were found to have strong EGFP fluorescence signals (Figure 4A). When comparing the fluorescence signals among the mouse lines, it is clear that high transgene copy number in the 3G line, indeed, provided an advantage to yield sufficient signal-to-noise fluorescence ratio. It is noted that the 16E line, despite harboring 10 transgene copies (Figure 1B), showed considerably weak EGFP fluorescence (Figure 4A). In the 16E line, the *EGFP* gene is located at downstream of the bicistronic mRNA that might affect the strength of IRES-dependent translation [30]. Organs of interest were examined (Figure 4B). In whole-mount specimen of the brain, the olfactory bulb located in the anterior part of the brain showed strong EGFP signals (Figure 4B, (a)). Mapping the expression pattern of ASS in the brain is a key to understanding its functional significance in the nervous system. We, therefore, studied *ASS-EGFP* expression in the brain as presented in Figure 5. On the other hand, the pancreas showed strong EGFP fluorescence signals in contrast to no signals in the spleen when examined macroscopically (Figure 4A and B, (b) (ii)). However, in frozen sections, spleen did show some signals (Figure 4B, (b) (iii)), while signals in the pancreas were mainly present in acinar cells (Figure 4B, (b) (iv)). Likewise, no apparent EGFP signal was detected in the heart and lung held in the thoracic cavity (Figure 4A), but EGFP signals appeared when frozen sections were examined (Figure 4B, (c)). The salivary gland showed strong fluorescence signals (Figure 4A). This structure in the rodent and in humans is composed of three pairs of glandular organs, i.e., parotid gland, sublingual gland and submandibular gland [31]. The signal was found predominantly in the sublingual gland (Figure 4B, (d)). The sublingual gland contains well-developed striated duct system that produces mucous secretion [31]. In whole-mount specimens of male urogenital organs, the prostate showed less pronounced signals in contrast to the kidney and the testis, which showed strong signals (Figure 4B, (e) (ii)). The testis is composed of seminiferous tubules that primarily contain germ cells and Sertoli cells, and interstitial tissues that mainly contain Leydig cells responsible for production and secretion of testosterone [32,33]. Strong EGFP expression was found in the interstitial tissue but not in seminiferous tubules (Figure 4B, (e) (iii) & (iv)). EGFP expression in the liver, kidney and intestine has previously been addressed [20] and is not further studied here.

*EGFP* mRNA expression in tissues of the transgenic mice was examined by northern blotting to detect the 1.0-kb transcript of the *Tg (ASS-Ex3-EGFP)* lines, and the 4.1- and 3.1-kb transcripts of the *Tg (ASS-Ex16-EGFP)* lines (Figure 4C, left panel). In both transgenic lines, the highest *EGFP* expression levels were detected in the liver and kidney. The relative *EGFP* RNA levels in the tissues analyzed were similar between *Tg (ASS-Ex3-EGFP)* and *Tg (ASS-Ex16-EGFP)* indicating that tissue-specific expression is mainly controlled by promoter activities. The *ASS* transgene in the transgenic model is of human origin. We showed that expression of the human *ASS* gene in the liver of the transgenic mice followed the mouse *Ass* developmental pattern. Conversely, the *EGFP* mRNA expression profile in the small intestine resembles the human *ASS* in that substantial levels of *EGFP* mRNA could still be detected at 8 weeks of age in contrast to mouse *Ass* mRNA that disappears at 3 weeks after birth [20]. Indeed, when the endogenous *Ass* mRNA levels were studied in wild-type mouse (Figure 4C, WT in left panel), the intestine showed relatively low expression whereas the liver and kidney showed the highest *Ass* mRNA levels, followed by that of the testis. Besides the intestine, relative low *Ass* mRNA level was found in the muscle when compared to that of *EGFP* mRNA in the transgenic mice. Whether this was due to the difference in ASS expression profiles between human and mouse as occurred in the intestine requires further studies. Furthermore, *EGFP* mRNA expression in the spleen was low in the transgenic lines in contrast to a substantial high *Ass* mRNA level in wild-type mouse. *Ass* mRNA was found at a low level in the rat spleen and increased markedly by bacterial lipopolysaccharide treatment [34]. Apparently, ASS expression level in the spleen is influenced by environmental conditions. On the other hand, to determine the relative ASS RNA levels of human and mouse, northern blot was performed in lines 16H, 16 F and 16E of *Tg (ASS-Ex16-EGFP)*, which carry 2, 5 and 10 transgene copies, respectively. The level of human *ASS* RNA was higher than that of mouse *Ass* RNA in multiple tissues examined (Figure 4C, right panel). Notably, the human *ASS* RNA in the liver was estimated to be 2-, 10- and 12-fold higher than that of the mouse RNA in lines 16H, 16 F and 16E, respectively. Since ASS is a potential limiting step in NO production [2], *Tg (ASS-Ex16-EGFP)* is particularly suitable for studying the effects of ASS overproduction on NO action during inflammation or disease progression.

### *ASS-EGFP* transgene expression in the brain of young mice

Localization of ASS in the brain has previously been studied by immunocytochemical methods and was found to be constitutively expressed in neurons and astrocytes [35,36]. During brain inflammation, ASS is also expressed in microglial cells [37]. Nitric oxide (NO), an important signaling molecule, is widely used in the nervous system [38]. Abnormal NO signaling is known to contribute to a variety of neurodegenerative pathologies [38]. Conceivably, ASS, acting as an integral part of the NO producing pathway, would affect the neuromodulatory system in the brain [2,38]. Moreover, in the brains of Alzheimer patients, ASS and inducible nitric oxide synthase are found to co-express in neurons and glial cells [36]. In addition to the function as a potential limiting step in NO production, ASS may have a direct role in neuromodulation. This is because the product of ASS, argininosuccinate, has been implicated as a putative neuromodulator [2,35]. Furthermore, viral encephalitis causes focal signs of cerebral dysfunction. A recent report by Grady *et al.* [39] provides evidences that ASS depletion produces a cellular metabolic state conducive to herpes simplex virus 1 infection. It is plausible that neurons in ASS-expressing brain regions might be susceptible to viral infection due to metabolic states that cause decreased ASS level. Therefore, localization of ASS expression in the brain is of great importance.

*ASS-EGFP* expression in the brain of 4 week-old *Tg (ASS-Ex3-EGFP) 3G* mice was examined. Under a fluorescence microscope, serial frozen coronal sections from the olfactory bulb to the cerebellum revealed specific brain areas that exhibited EGFP signals (Figure 5). Significant EGFP signals indicative of ASS expression is present in the olfactory bulb (Figure 5A), septum area (Figure 5B), habenular nucleus of thalamus (Figure 5C) and the choroid plexus of all ventricles (Figure 5D to 5F).

Mouse olfactory bulb, located at the most anterior part of the brain, is responsible for the sense of smell [40,41]. The bulb is divided into two distinct structures: the main olfactory bulb (MOB) and the accessory olfactory bulb (AOB) [40,41]. Both are multilayered structures, and in our transgenic mice showed significant EGFP expression in the granule cell layer (Gr), the glomerular layer (Gl) and ependyma cells lining the olfactory ventricle (Ep/OV). In contrast, the orbital cortex (OCTX) showed no detectable EGFP signals (Figure 5A). EGFP is expressed in cells of the olfactory bulb but not in other constituent parts related to smell, i.e., olfactory tubercle and piriform cortex, suggesting that the olfactory bulb might bear localized functions, a proposition that requires further investigation.

Septum, a structure of the limbic brain, has been implicated in cognition and emotion. This subcortical structure has rich connections with the hippocampus, amygdale and the thalamus, the major sensory relay station to and from the cerebral cortex [42,43]. Strong and clear EGFP signals were present in septal nuclei including lateral septal nucleus dorsal part (LSD), lateral septal nucleus intermediate part (LSI) and medial septal nucleus (MS) (Figure 5B). Expression of nitric oxide synthase in the septum and that of NO in the septohippocampal cholinergic system have been implicated to control the generation of theta (θ) wave, which is linked to mechanisms of learning and memory [44]. The septum also serves as a reward center by inhibiting negative emotions and expressing the pleasurable sensation *via* inhibitory signals sent to amygdale [45]. Lower cell density in septal nuclei has been reported in patients with mental disorders such as bipolar disorder, major depressive disorder and schizophrenia [46], further supporting the notion that septal nuclei are important in emotional regulation. The intense EGFP expression in the septum, a brain area endowed with so many important functions, makes the delineation of the role of ASS in each nuclei with their respective connecting brain areas a challenge in future studies.

EGFP expression was also detected in the habenular nuclei of the thalamus (Figure 5C). This medial-located pair of nuclei and the pineal body constitutes the epiphysis which, together with the suprachiasmatic nucleus (SCN) of the hypothalamus, is responsible for the circadian control of behavior such as locomotor rhythm [47]. More recently, habenula has been identified as the key brain structure responsible for stress evasion and value-based decision making [48]. Recent evidence also indicated that altered basal ganglia activity leads to lateral habenula hyperactivity, which in turn down-regulates the serotonergic system, resulting in depressive symptoms in patients with Parkinson’s disease [49].

The choroid plexus (CP) located in the lateral ventricle (Figure 5D) the third ventricle (Figure 5E) and the fourth ventricle (Figure 5F) all exhibited strong EGFP signals. The choroid plexus is a highly vascularized tissue located in each ventricle of the brain known for generating the cerebrospinal fluid (CSF) [50]. Recent progresses have shown that CP and the instructive signals in the CSF may provide means for regulating brain structure and function in health and in diseases [51]. In particular, choroid plexus may release into CSF morphogens EGF, FGF-2 and retinoic acid, which are keys to adult neurogenesis [51]. Choroid plexus is also the source of phosphorylated Tau and Aβ in CSF, the ratio of the two may serve as an important index for detecting, diagnosing and treating Alzheimer disease in the aging brain [51].

It would also be interesting to learn the similarities and differences of ASS expression patterns between brains of primates and rodents. The functional significance of the differences, if any, can conceivably be explored by using the mouse as an experimental model [52]. Nevertheless, *Ass* brain profile using the BAC transgenic reporter is not yet available in Gene Expression Nervous System Atlas (GENSAT) [53] to allow comparison of ASS expression patterns across species.

### Down-regulated EGFP expression in hepatocellular carcinoma (HCC) in the *Tg (ASS- EGFP)* transgenic mice

**Figure 6:**
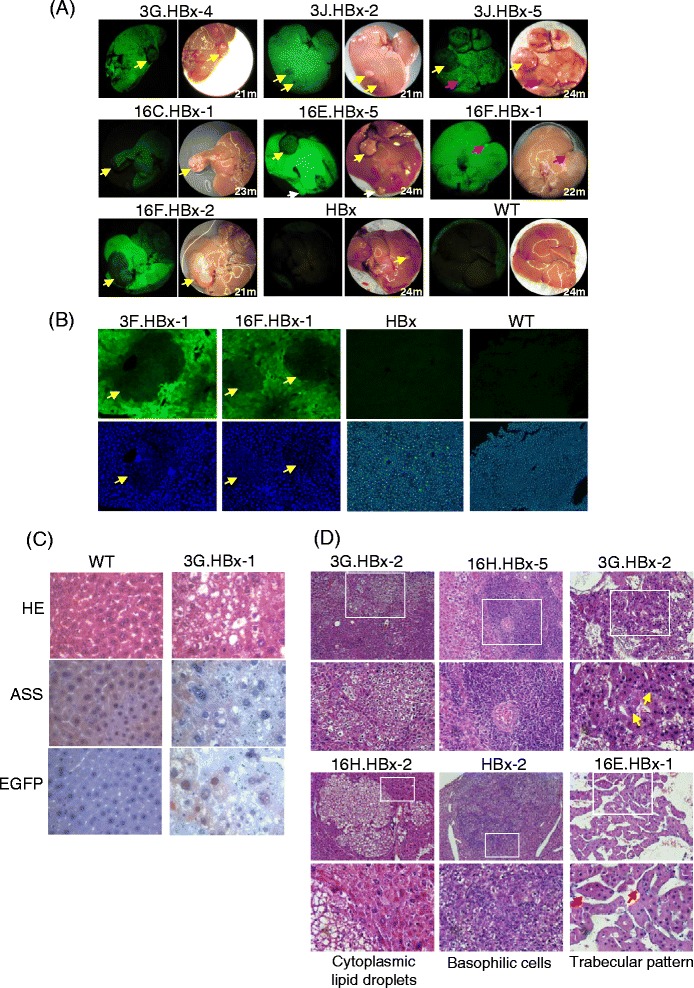
**Down-regulated** ***ASS-EGFP*** **expression in** ***HBx*** **-induced hepatocellular carcinoma in** ***Tg (ASS-EGFP, HBx)*** **double transgenic mice.** In all subfigures, mice are denoted by the *ASS-EGFP* designations as in Figure 1, followed by *HBx* to indicate double transgenics; the numerical after *HBx* denotes the specific mouse analyzed. The 16C line used in 16C. HBx-1 (A) and 3 F line in 3 F. HBx-1 (B) carry 3 and 2 transgene copies, respectively. **(A)** Localization of EGFP levels in tumor nodules observed under a fluorescence dissecting microscope. The age of the mice in months (m) when sacrificed is shown in the bottom right corner of each image. Yellow and pink arrows indicate reduced and enhanced EGFP fluorescence, respectively; the white arrow indicates apparently unaltered fluorescence levels compared with the surrounding tissue. *HBx* was single transgenic mouse; WT, a non-transgenic control. (**B)** Correlation of tumor morphology and EGFP expression. Liver frozen sections were taken from tumors of the transgenic lines. WT was a non-tumorous section from a normal mouse used as a control. Top panels, fluorescence sections; bottom panels, DAPI-stained sections. Yellow arrows indicate sites of down-regulated EGFP expression. (**C)** Immunohistochemical analysis of ASS and EGFP expression in tumors of the *Tg (ASS-EGFP, HBx)* double transgenic mice. Tumor tissues were reacted with an anti-ASS or anti-EGFP antibody and incubated with the biotinylated secondary antibody coupled with streptavidin conjugated-HRP. HRP activities were visualized by the application of chromogen DAB to produce brown coloration while the slides were counterstained with hematoxylin. (**D)** Histochemical analysis of selected tumor morphology of the double transgenic mice. After HE staining, the tissue samples were observed under a light microscope. Boxed field in the top panel is magnified and is shown in the bottom panel. Yellow arrows indicate central-sinusoidal trabecular pattern; red arrows indicate peripheral-sinusoidal trabecular pattern.

Loss of ASS function has been reported in cancer cells, including HCC [54,55]. In this work, transgenic mice harboring the *EGFP* reporter driven by *ASS* regulatory elements were used to examine correlation between HCC tumorigenesis and *ASS* expression. To generate HCC mice, *Tg (ASS-Ex3-EGFP)* and *Tg (ASS-Ex16-EGFP)* mice were crossed with transgenic mice harboring the *HBx* gene of the hepatitis B virus [22] to produce *Tg (ASS-EGFP, HBx)* double transgenic mice. The mice were kept in specific pathogen-free conditions. At about 24 months of age, the mice were sacrificed, and the livers were removed for analysis. The weight of the liver and the bodyweight of each double transgenic mouse were determined as an indication of hepatomegaly. The liver-to- bodyweight mean weight ratio of the double transgenic mice was 0.070 ± 0.031, and was significantly higher than that of non-transgenic mice of the same age and strain, which was 0.040 ± 0.003 (*p* < 0.05, Student *t* test). The results clearly indicated hepatomegaly in the double transgenic mice, in which HCC nodules were, indeed, observed in the liver (Figure 6).

At about 24 months of age, the mice were sacrificed and the livers were removed for analysis. When examined under a fluorescence dissecting microscope, EGFP fluorescence in the tumor sites was significantly lower in intensity than that of the surrounding non-tumorous tissues in both *Tg (ASS-Ex3-EGFP)*- and *Tg (ASS-Ex16-EGFP)*-derived *HBx* double transgenic mice (Figure 6A, yellow arrows). The results indicate down-regulated transcriptional activities of the *ASS* regulatory elements in driving the expression of the *EGFP* transgene, consistent with down-regulated *ASS* expression in HCC in human subjects [16]. It was noted that a minority of the tumors showed enhanced (Figure 6A, pink arrows) or no change (Figure 6A, white arrow) in EGFP fluorescence intensity, suggesting possible microenvironmental influences on the *ASS* promoter activities in the transgenic mice.

To examine the morphology of the HCC developed in the double transgenic mice, frozen HCC sections were examined (Figure 6B). The results again showed decreased fluorescence intensity in most tumor nodules in both the *Tg (ASS-Ex3-EGFP)*- and *Tg (ASS-Ex16-EGFP)*-derived *HBx* double transgenic lines. On DAPI staining, nuclei in the tumor tissues were smaller and denser, and were morphologically distinctive to the surrounding structure of the liver (Figure 6B). Immunohistochemical studies were also performed to demonstrate that ASS and EGFP proteins indeed decreased in the tumor nodules (Figure 6C). Moreover, when HE-stained paraffin-embedded tissue sections were examined, typical HCC morphologies including cytoplasmic lipid droplets, basophilic cells and trabecular patterns were observed (Figure 6D). In the HCC, there were relatively more cells with cytoplasmic lipid droplets (Figure 6D, left panels), consistent with frequent association of fatty liver with HCC. The morphology of the basophilic cells was similar to the DAPI-stained morphology of frozen sections of the HCC nodules (Figure 6B) in being numerous and dense and in having smaller nuclei (Figure 6D, middle panels). In well-differentiated HCC, the regularly-seen trabecular pattern is HCC intersperses with sinusoids. HCC trabecular patterns are divided into two morphological types: central-sinusoidal and peripheral-sinusoidal pattern [56]. In the central-sinusoidal pattern of the double transgenic mice, sinusoids were narrower and were branched, and formed multi-layered HCC morphology (Figure 6D, yellow arrows). In the peripheral-sinusoidal pattern, sinusoids were wider and the HCC cells were enclosed by endothelium to form multiple layers of trabeculae, which were separated (Figure 6D, red arrows). Taken together, the *HBx* double transgenic mice developed histologically-verified HCC in which *ASS* expression was down-regulated, strongly supporting close resemblance of the transgenic mouse model with the clinical patterns of *ASS* expression in cancers. Moreover, down-expression of ASS occurred in HCC from both the *Tg (ASS-Ex3-EGFP)*- and *Tg (ASS-Ex16-EGFP)*-derived HBx double transgenic lines, suggesting that mechanism (s) affecting promoter activity are involved.

The transgenic mouse model documented in this study may also be exploited for prostate, pancreatic, renal cancers and melanoma that show down-regulated *ASS* expression [13–15], especially when EGFP was shown to be expressed in these tissues in the *Tg (ASS-EGFP)* mice (Figure 4). Conceivably, using our mouse model, epigenetic regulation of *ASS* transcription and drug or immunological evasion in treating cancers may be investigated and the level of EGFP fluorescence can be used as a direct monitor of the effects.

## Conclusions

We report in this work the development of two *ASS-EGFP* transgenic mouse lines based on the use of modified BAC cassettes: the transcription reporter mice *Tg (ASS-Ex3-EGFP)* and the transcription/post-transcription couple reporter mice *Tg (ASS-Ex16-EGFP)*. These two transgenic lines permit temporal and spatial expression profiling of *ASS* gene expression, thus, contributing insights to regulated *ASS* expression. Particularly, by comparing the expression profiles between these two lines, signals and the regulatory elements involved in novel regulation of *ASS* precursor RNA stability may be deduced. Moreover, the ASS expression profile in the brain should provide appropriate paths to pursuit functional roles of ASS in the nervous system. In this respect, the *Tg (ASS-Ex16-EGFP)* mouse line is highly suitable for examination of the influence of human ASS expression on mouse behavior upon inflammation or aging. Likewise, effects of ASS overexpression on the development of HCC in double transgenic mice of *Tg (ASS-Ex16-EGFP, HBx)* at resting state and upon exogenous insults, such as virus infection or inflammation, can further be analyzed using this transgenic mouse system.

## References

[1] Morris SM (2006). Arginine: beyond protein. Am J Clin Nutr.

[2] Husson A, Brasse-Lagnel C, Fairand A, Renouf S, Lavoinne A (2003). Argininosuccinate synthetase from the urea cycle to the citrulline-NO cycle. Eur J Biochem.

[3] Haines RJ, Pendleton LC, Eichler DC (2011). Argininosuccinate synthase: at the center of arginine metabolism. Int J Biochem Mol Biol.

[4] Guei TR, Liu MC, Yang CP, Su TS (2008). Identification of a liver-specific cAMP response element in the human argininosuccinate synthetase gene. Biochem Biophys Res Commun.

[5] Brasse-Lagnel C, Lavoinne A, Loeber D, Fairand A, Bole-Feysot C, Deniel N (2007). Glutamine and interleukin-1beta interact at the level of Sp1 and nuclear factor-kappaB to regulate argininosuccinate synthetase gene expression. FEBS J.

[6] Tsai WB, Aiba I, Lee SY, Feun L, Savaraj N, Kuo MT (2009). Resistance to arginine deiminase treatment in melanoma cells is associated with induced argininosuccinate synthetase expression involving c-Myc/HIF-1alpha/Sp4. Mol Cancer Ther.

[7] Su TS, Beaudet AL, O’Brien WE (1981). Increased translatable messenger ribonucleic acid for argininosuccinate synthetase in canavanine-resistant human cells. Biochemistry.

[8] Tsai TF, Su TS (1995). A nuclear post-transcriptional event responsible for overproduction of argininosuccinate synthetase in a canavanine-resistant variant of a human epithelial cell line. Eur J Biochem.

[9] Tseng SH, Cheng CY, Huang MZ, Chung MY, Su TS (2013). Modulation of formation of the 3′-end of the human argininosuccinate synthetase mRNA by GT-repeat polymorphism. Int J Biochem Mol Biol.

[10] Lavoinne A, Meisse D, Quillard M, Husson A, Renouf S, Yassad A (1998). Glutamine and regulation of gene expression in rat hepatocytes: the role of cell swelling. Biochimie.

[11] Schimke RT (1964). Enzymes of arginine metabolism in mammalian cell culture. i. repression of argininosuccinate synthetase and argininosuccinase. J Biol Chem.

[12] Su TS, Bock HG, O’Brien WE, Beaudet AL (1981). Cloning of cDNA for argininosuccinate synthetase mRNA and study of enzyme overproduction in a human cell line. J Biol Chem.

[13] Dillon BJ, Prieto VG, Curley SA, Ensor CM, Holtsberg FW, Bomalaski JS (2004). Incidence and distribution of argininosuccinate synthetase deficiency in human cancers: a method for identifying cancers sensitive to arginine deprivation. Cancer.

[14] Delage B, Fennell DA, Nicholson L, McNeish I, Lemoine NR, Crook T (2010). Arginine deprivation and argininosuccinate synthetase expression in the treatment of cancer. Int J Cancer.

[15] Phillips MM, Sheaff MT, Szlosarek PW (2013). Targeting arginine-dependent cancers with arginine-degrading enzymes: opportunities and challenges. Cancer Res Treat.

[16] Yang H, Lin M, Xiong FX, Yang Y, Nie X, Zhou RL (2010). Reduced expression of ASS is closely related to clinicopathological features and post-resectional survival of hepatocellular carcinoma. Oncol Lett.

[17] Wu L, Li L, Meng S, Qi R, Mao Z, Lin M (2013). Expression of argininosuccinate synthetase in patients with hepatocellular carcinoma. J Gastroenterol Hepatol.

[18] Szlosarek PW, Klabatsa A, Pallaska A, Sheaff M, Smith P, Crook T (2006). In vivo loss of expression of argininosuccinate synthetase in malignant pleural mesothelioma is a biomarker for susceptibility to arginine depletion. Clin Cancer Res.

[19] Szlosarek PW, Grimshaw MJ, Wilbanks GD, Hagemann T, Wilson JL, Burke F (2007). Aberrant regulation of argininosuccinate synthetase by TNF-alpha in human epithelial ovarian cancer. Int J Cancer.

[20] Shiue SC, Huang MZ, Su TS (2014). A transgenic approach to study argininosuccinate synthetase gene expression. J Biomed Sci.

[21] Gust B, Challis GL, Fowler K, Kieser T, Chater KF (2003). PCR-targeted Streptomyces gene replacement identifies a protein domain needed for biosynthesis of the sesquiterpene soil odor geosmin. Proc Natl Acad Sci U S A.

[22] Wu BK, Li CC, Chen HJ, Chang JL, Jeng KS, Chou CK (2006). Blocking of G1/S transition and cell death in the regenerating liver of Hepatitis B virus X protein transgenic mice. Biochem Biophys Res Commun.

[23] Gong S, Zheng C, Doughty ML, Losos K, Didkovsky N, Schambra UB (2003). A gene expression atlas of the central nervous system based on bacterial artificial chromosomes. Nature.

[24] Ko MS, Kitchen JR, Wang X, Threat TA, Wang X, Hasegawa A (2000). Large-scale cDNA analysis reveals phased gene expression patterns during preimplantation mouse development. Development.

[25] Mouse Genome Informatics [http://www.informatics.jax.org/searches/probe.cgi?824472]

[26] GenePaint.org [http://www.genepaint.org/Midline.htm]

[27] Kaufman MH (1992). The Atlas of Mouse Development.

[28] The Mouse Gene Expression Database (GXD) [http://www.informatics.jax.org/gxd/marker/MGI:88090]

[29] Noctor SC, Martínez-Cerdeño V, Ivic L, Kriegstein AR (2004). Cortical neurons arise in symmetric and asymmetric division zones and migrate through specific phases. Nat Neurosci.

[30] Hennecke M, Kwissa M, Metzger K, Oumard A, Kröger A, Schirmbeck R (2001). Composition and arrangement of genes define the strength of IRES-driven translation in bicistronic mRNAs. Nucleic Acids Res.

[31] Shackleford JM, Schneyer CA (1964). Structural and functional aspects of rodent salivary glands including two desert species. Am J Anat.

[32] Maekawa M, Kamimura K, Nagano T (1996). Peritubular myoid cells in the testis: their structure and function. Arch Histol Cytol.

[33] Haider SG (2004). Cell biology of Leydig cells in the testis. Int Rev Cytol.

[34] Nagasaki A, Gotoh T, Takeya M, Yu Y, Takiguchi M, Matsuzaki H (1996). Coinduction of nitric oxide synthase, argininosuccinate synthetase, and argininosuccinate lyase in lipopolysaccharide-treated rats. RNA blot, immunoblot, and immunohistochemical analyses. J Biol Chem.

[35] Nakamura H, Saheki T, Ichiki H, Nakata K, Nakagawa S (1991). Immunocytochemical localization of argininosuccinate synthetase in the rat brain. J Comp Neurol.

[36] Heneka MT, Wiesinger H, Dumitrescu-Ozimek L, Riederer P, Feinstein DL, Klockgether T (2001). Neuronal and glial coexpression of argininosuccinate synthetase and inducible nitric oxide synthase in Alzheimer disease. J Neuropathol Exp Neurol.

[37] Heneka MT, Schmidlin A, Wiesinger H (1999). Induction of argininosuccinate synthetase in rat brain glial cells after striatal microinjection of immunostimulants. J Cereb Blood Flow Metab.

[38] Wiesinger H (2001). Arginine metabolism and the synthesis of nitric oxide in the nervous system. Prog Neurobiol.

[39] Grady SL, Purdy JG, Rabinowitz JD, Shenk T (2013). Argininosuccinate synthetase 1 depletion produces a metabolic state conducive to herpes simplex virus 1 infection. Proc Natl Acad Sci U S A.

[40] Yokosuka M (2012). Histological properties of the glomerular layer in the mouse accessory olfactory bulb. Exp Anim.

[41] Pain F, L’heureux B, Gurden H (2011). Visualizing odor representation in the brain: a review of imaging techniques for the mapping of sensory activity in the olfactory glomeruli. Cell Mol Life Sci.

[42] Gray JA, McNaughton N (1983). Comparison between the behavioural effects of septal and hippocampal lesions: A review. Neurosci Biobehav Rev.

[43] Calandreau L, Jaffard R, line Desmedt A (2007). Dissociated roles for the lateral and medial septum in elemental and contextual fear conditioning. Learn Mem.

[44] Hasselmo ME (2005). What is the function of hippocampal theta rhythm?–Linking behavioral data to phasic properties of field potential and unit recording data. Hippocampus.

[45] Risold PY, Paxinos G (2004). The septal region. The Rat Nervous System. Chapter 20.

[46] Leveque M, Leveque M (2014). The neuroanatomy of emotions. Psychosurgery: New Techniques for Brain Disorders. Chapter 2.

[47] Hikosaka O (2007). Habenula. Scholarpedia.

[48] Hikosaka O (2010). The habenula: from stress evasion to value-based decision-making. Nat Rev Neurosci.

[49] Sourani D, Eitan R, Gordon N, Goelman G (2012). The habenula couples the dopaminergic and the serotonergic systems: application to depression in Parkinson’s disease. Eur J Neurosci.

[50] Strazielle N, Ghersi-Egea JF (2000). Choroid plexus in the central nervous system: biology and physiopathology. J Neuropathol Exp Neurol.

[51] Lehtinen MK, Bjornsson CS, Dymecki SM, Gilbertson RJ, Holtzman DM, Monuki ES (2013). The choroid plexus and cerebrospinal fluid: emerging roles in development, disease, and therapy. J Neurosci.

[52] Greely HT, Cho MK, Hogle LF, Satz DM (2007). Thinking about the human neuron mouse. Am J Bioeth.

[53] The Gene Expression Nervous System Atlas (GENSAT) [http://www.gensat.org/index.html] 10.1038/nn0504-483 15114362

[54] Cheng PN, Lam TL, Lam WM, Tsui SM, Cheng AW, Lo WH (2007). Pegylated recombinant human arginase (rhArg-peg5,000mw) inhibits the in vitro and in vivo proliferation of human hepatocellular carcinoma through arginine depletion. Cancer Res.

[55] Feun L, You M, Wu CJ, Kuo MT, Wangpaichitr M, Spector S (2008). Arginine deprivation as a targeted therapy for cancer. Curr Pharm Des.

[56] Kung IT, Chan SK, Fung KH (1991). Fine-needle aspiration in hepatocellular carcinoma. Combined cytologic and histologic approach. Cancer.

